# [(2-Dimesitylboryl)phenyl]ethynyl-Substituted [2.2]Paracyclophane Exhibiting Circularly Polarized Luminescence in Both Solution and Solid-State

**DOI:** 10.3390/molecules30020390

**Published:** 2025-01-18

**Authors:** Lianfeng Guo, Mengyuan Zhang, Cuihua Zhao

**Affiliations:** 1 School of Chemistry and Chemical Engineering, Shandong University, Jinan 250100, China; 202112183@mail.sdu.edu.cn; 2 College of Chemical Engineering and Environmental Engineering, Weifang University, Weifang 261061, China

**Keywords:** [2.2]paracyclophane, triarylborane, circularly polarized luminescence, high emission efficiency, high luminescence dissymmetry factor

## Abstract

Developing a new type of circularly polarized luminescent active small organic molecule that combines high fluorescence quantum yield and luminescence dissymmetric factor in both solution and solid state is highly challenging but promising. In this context, we designed and synthesized a unique triarylborane-based [2.2]paracyclophane derivative, ***m*-BPhANPh_2_-Cp**, in which an electron-accepting [(2-dimesitylboryl)phenyl]ethynyl group and an electron-donating *N,N*-diphenylamino group are introduced into two different benzene rings of [2.2]paracyclophane. Owing to the electronic effect of these two substituents, this compound can display charge-transfer emission with large Stokes shifts (∆*υ* = 4.23 − 8.20 × 10^3^ cm^−1^) and fair quantum yields (*Φ*_F_ = 0.15 − 0.37) in solutions. In addition, this compound can emit strong blue fluorescence in the solid state with quantum yields that are even much higher than in solution (*Φ*_F_ up to 0.64 in powder and spin-coated film). Moreover, the enantiomeric forms of ***m*-BPhANPh_2_-Cp** can show strong CPL signals in both dilute solution and solid state with |*g*_lum_|s up to 9.6 × 10^−3^ and 5.4 × 10^−3^, respectively. Thus, it is possible to achieve tunable CPL from blue to yellow in solution with high *B*_CPL_s ranging from 56.7 to 26.6 M^−1^ cm^−1^ and intense blue CPL combing high *Φ*_F_ and |*g*_lum_| in the solid state.

## 1. Introduction

Circularly polarized luminescence (CPL) refers to the differential emission between left- and right-handed circularly polarized light from chiral materials or achiral materials in chiral environments. In recent years, CPL-active materials have received widespread attention due to their potential applications in 3D displays [1,2,3], optical information storage and processing [4,5], CPL lasers [6,7], CPL sensors [8,9,10], biological probes [11,12], and asymmetric photosynthesis [13,14]. Unlike circular dichroism (CD) with differential absorption of left- and right-handed circularly polarized light, which can be used to obtain structural information of the ground state, CPL spectra directly reflect the structural information of the excited state of chiral compounds. Fluorescence quantum yield (*Φ*_F_) and luminescence dissymmetry factor (*g*_lum_) are two key parameters to evaluate CPL property. *Φ*_F_ reflects the fluorescence efficiency. *g*_lum_ shows the degree of dissymmetry and is defined as 2(*I*_L_ − *I*_R_)/(*I*_L_ + *I*_R_), where *I*_L_ and *I*_R_ are the luminescence intensity of left- and right-handed circularly polarized lights, respectively. These two parameters, together with the molar extinction coefficient (*ε*), define CPL brightness (*B*_CPL_ = *ε* × *Φ*_F_ × |*g*_lum_|/2), which has more recently been introduced to evaluate the overall performance of CPL property [15,16].

Compared with the traditional chiral transition-metal complexes [17,18] and chiral polymers [19,20], small organic molecules generally have distinct advantages, such as higher emission efficiency, exact molecular structure, facile modification of chemical structure, and easy fabrication [21,22,23,24]. Especially, the facile structural modification permits easy tuning of not only emission wavelengths but also crucial CPL property parameters, like *ε*, *Φ*_F_, |*g*_lum_| and thus *B*_CPL_. Therefore, the development of organic small molecule CPL-active materials is of great significance for constructing high-performance asymmetric light-emitting devices. However, currently, these materials face two crucial issues. Firstly, CPL-active small organic molecules (CPL-SOMs) tend to achieve high emission efficiency in solution but are accompanied by small |*g*_lum_|, ranging from 10^−3^ to 10^−5^ [25,26,27,28]. In addition, CPL-SOMs often suffered from aggregation-caused quenching (ACQ) effect due to intermolecular π–π stacking interactions, resulting in apparent or complete fluorescence quenching in the aggregated state [29,30]. Both of these phenomena lead to weak CPL signals, particularly in the solid state, which greatly limits their practical application in optoelectronic materials. Therefore, the design and synthesis of a class of CPL-SOMs combing high *Φ*_F_s and |*g*_lum_|s in both solution and solid state is challenging but promising [31,32,33,34].

**Figure 1:**
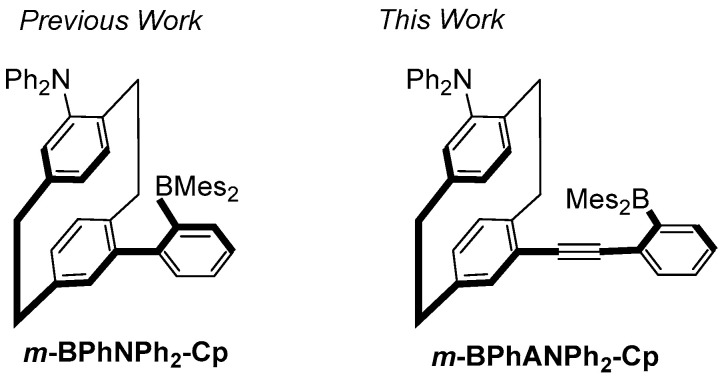
Chemical structure of 2-(dimesitylboryl)phenyl-substituted and [(2-dimethylboryl) phenyl]ethynyl-substituted [2.2]paracyclophanes.

To construct CPL-SOMs, [2.2]paracyclophane is one attractive scaffold due to its stable planar chirality and efficiency through space conjugation between two face-to-face benzene rings as the result of the short distance between them [35,36,37,38,39]. On the other hand, triarylboranes can serve as a unique bulky electron-accepting group due to the electronic effect of the vacant 2p orbitals of B element and the bulk steric effect of the aryl group to protect the B center. It has been well demonstrated that the introduction of the boryl group into the π-system containing electron-donating group to construct charge transfer (CT) emitting systems is effective in achieving intense fluorescence in both solution and solid state [40,41]. Therefore, introducing electron-accepting boryl and electron-donating groups into two different benzene rings of the [2.2]paracyclophane is expected to obtain CPL-SOMs with excellent luminescent properties [42,43,44]. Along this line, we recently reported a CT-emitting triarylborane-based [2.2]paracyclohexane, ***m*-BPhNPh_2_-Cp** [43] (Figure 1), in which 2-(dimesitylboryl)phenyl group and an electron-donating *N*,*N*-diphenylamino group were attached to the pseudo-*meta* positions of [2.2]paracyclophane. This molecule not only shows strong CPL with high *Φ*_F_s and |*g*_lum_|s in dilute solution but also exhibits solvent-driven sign inversion through modulation of excited-state dynamics between the localized excited state (LE) and CT state. The *Φ*_F_ and |*g*_lum_|s of ***m*-BPhNPh_2_-Cp** are high up to 0.82 and 1.67 × 10^−2^, respectively. Regretfully, *Φ*_F_ of ***m*-BPhNPh_2_-Cp** in the solid state is not very high (*Φ*_F_ = 0.19 in powder). In this context, we designed and synthesized a new pseudo-*meta*-substituted [2.2]paracyclophane derivative, ***m*-BPhANPh_2_-Cp** (Figure 1). Compared with ***m*-BPhNPh_2_-Cp**, ***m*-BPhANPh_2_-Cp** was modified by replacing the 2-(dimesitylboryl)phenyl with [(2-dimethylboryl)phenyl]ethynyl as the electron-accepting group. The achiral optical and chiroptical properties of ***m*-BPhANPh_2_-Cp** were investigated comprehensively in both solution and solid state. It was found that this compound can show particularly strong fluorescence in the solid state (*Φ*_F_ = 0.64 in powder and film), although the fluorescence in solutions is moderately strong (*Φ*_F_ = 0.15–0.37). More importantly, strong CPL signals were observed in both solution and solid state with high |*g*_lum_|s, which are high up to 9.62 × 10^−3^ and 5.40 × 10^−3^, respectively. In addition, theoretical calculations, including geometry optimizations in both the ground state (S_0_) and the first excited state (S_1_) and the corresponding vertical excitations, were also carried out to understand the optical properties of this compound. Herein, we report detailed results about synthesis, achiral optical properties, chiroptical properties, and theoretical calculations of ***m*-BPhANPh_2_-Cp**.

## 2. Results and Discussion

**Scheme 1:**
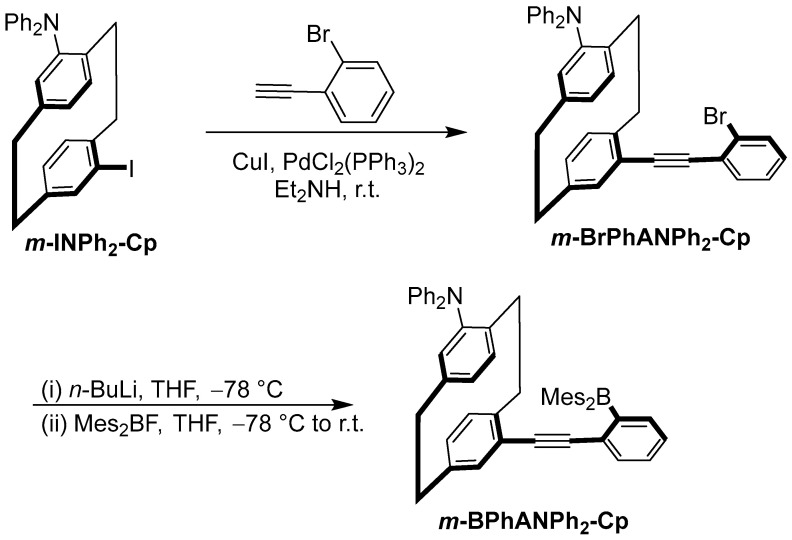
Synthesis of ***m*-BPhANPh_2_-Cp**.

The synthesis of the targe molecule ***m*-BPhANPh_2_-Cp** is shown in Scheme 1. It was prepared via the facile borylation of the bromide precursor, the pseudo-*meta* (2-bromophenyl)ethynyl- and *N,N*-diphenylamino-substituted [2.2]paracyclophane ***m*-BrPhANPh_2_-Cp**, through lithiation with *n*-BuLi and subsequent quenching with dimesitylboron fluoride. And the bromide precursor ***m*-BrPhANPh_2_-Cp** was obtained by the Sonogashira coupling of (2-bromophenyl)acetylene with pseudo-*meta* iodo- and *N,N*-diphenylamino-substituted [2.2]paracyclophane ***m*-INPh_2_-Cp**, which was prepared previously [43]. ***m*-BPhANPh_2_-Cp** is stable in air and water and can be purified by flash column chromatography. Its molecular structure was fully characterized by ^1^H NMR, ^13^C NMR (Supporting Information), and high-resolution mass spectrometry.

**Figure 2:**
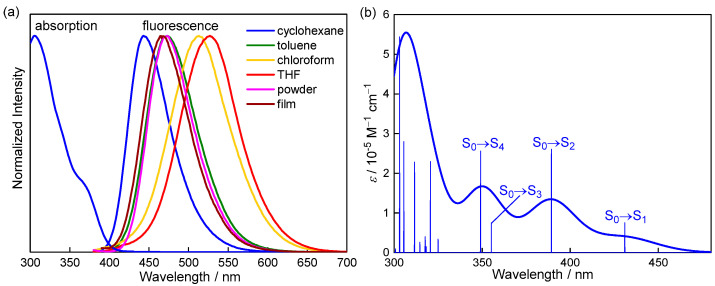
(**a**) Absorption spectra in cyclohexane and fluorescence spectra (*λ*_ex_ = 380 nm) in various solvents and solid state, and (**b**) calculated UV−vis absorption spectra in the optimized S_0_ geometry (calculated at the PBE0/6-31G(d) level) for ***m*-BPhANPh_2_-Cp**.

Figure 2 shows the UV-vis absorption spectra of ***m*-BPhANPh_2_-Cp** in cyclohexane and the fluorescence spectra in various solvents. The corresponding data are summarized in Table 1. In cyclohexane, the longest wavelength absorption band was observed as a shoulder band at 373 nm with moderate intensity (*ε* = 0.91 × 10^4^ M^–1^ cm^–1^). The maximum absorption peak was located at 305 nm (*ε* = 3.19 × 10^4^ M^–1^ cm^–1^). Regarding the emission, ***m*-BPhANPh_2_-Cp** displays sky blue fluorescence at 443 nm with fairly good efficiency (*Φ*_F_ = 0.37). It was noted there was almost no overlap between the absorption and emission spectra due to the large Stokes shift (∆*ν* = 4.23 × 10^3^ cm^–1^). As for the photophysical properties, another notable thing was the significant fluorescence solvatochromism. The fluorescence was red-shifted by 84 nm from cyclohexane to THF as the solvent polarity increased. As a consequence, ***m*-BPhANPh_2_-Cp** was a yellow emissive in THF with the maximum wavelength at 527 nm. Although *Φ*_F_ decreases with the increasing solvent polarity, this molecule can remain moderately fluorescent even in highly polar THF with a *Φ*_F_ of 0.15. In contrast to the significant fluorescence solvatochromism, no significant solvation effect was observed in the absorption. The significant fluorescence solvatochromism clearly suggests a more polarized structure in the excited state than in the ground state, which is consistent with the CT nature of the S_1_ state due to the D-π-A structure of **m-BPhANPh_2_-Cp** [45,46,47,48,49].

**Figure 3:**
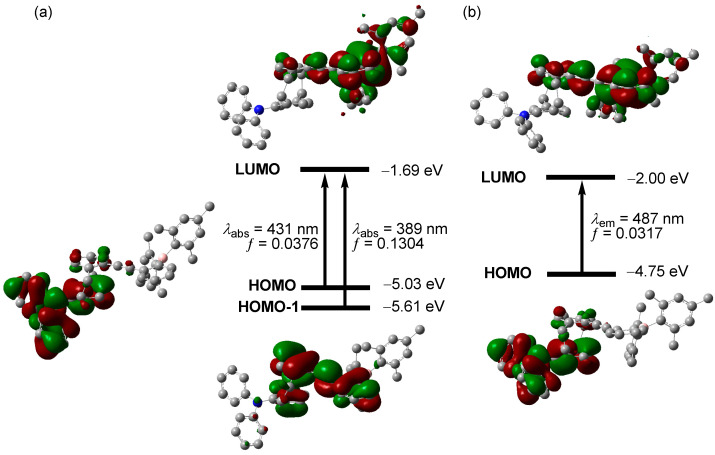
(**a**) Kohn−Sham energy levels, pictorial drawing of frontier orbitals, and transitions of ***m*-BPhANPh_2_-Cp** in the S_0_ state, and (**b**) in the S_1_ state, calculated at the TD/PBE0/6-31G(d) level of theory.

To shed light on the photophysical properties, we carried out comprehensive theoretical calculations, including the geometry optimizations in S_0_ and S_1_ states and the corresponding TD-DFT calculations. In the S_0_ state, the LUMO of ***m*-BPhANPh_2_-Cp** was mainly localized on the [2-(dimesitylboryl)phenyl]ethynyl group and its attached phenyl ring of the [2.2]paracyclophane while the HOMO was mainly distributed on the N,N-diphenylamino and [2.2]paracyclophane (Figure 3). The S_0_→S_1_ excitation was assignable to HOMO→LUMO transition, the oscillator strength of which was very low (*f* = 0.0376). Apparently, S_1_ was characterized by the CT nature, which again proves the effectiveness through space CT in [2.2]paracyclophanes modified with electron donating and accepting groups at two different rings. A careful comparison between the experiment and theoretically simulated absorption spectra found that the longest wavelength absorption band, which was observed as a shoulder band, actually corresponded to S_0_→S_2_ excitation, the oscillator strength of which was much higher (*f* = 0.1304). The S_0_→S_2_ excitation of ***m*-BPhANPh_2_-Cp** was assigned to the HOMO–1→LUMO transition with HOMO–1 spreading over the [2.2]paracyclophane and phenylethynyl moiety. Therefore, the S_2_ state was more like a LE state without the involvement of the amino group. Probably due to the very low transition probability of S_0_→S_1_ excitation, the corresponding absorption band was immersed into the tail of the absorption band arising from S_0_→S_1_ excitation and, thus, was hardly detected. Although the transition probability for the excitation to the S_2_ state was much higher than the excitation to the S_1_ state, the fluorescence locations and significant solvatochromism of ***m*-BPhANPh_2_-Cp** clearly indicate the great involvement of the amino group in its fluorescence and thus exclude the possibility for the fluorescence to arise from deactivation of the S_2_ state [44,50]. From the S_0_ to S_1_ state, no great changes were observed for the electronic distribution of HOMO and LUMO; the HOMO (Δ*E* = 0.28 eV) and LUMO (Δ*E* = 0.31 eV) energy levels changed a lot, which may have been caused by the great structural relaxation in the excited state. It is obvious that there was only a very small overlap between HOMO and LUMO in both S_0_ and S_1_ geometries, which confirmed the efficient through space CT nature of the S_1_ state. Although the theoretical excitation energies are underestimated to some extent, the calculation results clearly demonstrate the through space CT characteristic of S_1_ for ***m*-BPhANPh_2_-Cp**, which agrees well with its large Stokes shift and significant fluorescence solvatochromism.

It is worth noting that ***m*-BPhANPh_2_-Cp** is highly emissive in the solid state. Its solid emission properties were herein characterized in both powder and film forms (Figure 2, Table 1). The fluorescence properties are very close between the powder and film states with identical *Φ*_F_ (0.64) and a slight difference in maximum wavelength (Δ*λ* = 8 nm). The *Φ*_F_ in the solid state is significantly higher even than that in cyclohexane and is also unusually high in small organic molecules. Hence, both the powder and film of ***m*-BPhANPh_2_-Cp** emit intense blue fluorescence with maximum fluorescence wavelengths located at 473 nm and 465 nm, respectively. The emission positions of powder and film are similar to that in the toluene solution, indicating a similar environment between the solid state and toluene solution, proving the absence of intermolecular π–π interaction in the solid state. The absence of intermolecular π–π interaction in the solid state may be due to the strong steric effect of *N,N*-diphenylamino, and BMes_2_ groups, which can effectively avoid the ACQ effect. In order to further elucidate the emission properties of ***m*-BPhANPh_2_-Cp** in the solid state, the fluorescence lifetime was measured, and the radiative decay rate constant (*k*_r_) and nonradiative decay rate constant (*k*_nr_) were calculated. The fluorescence lifetimes in powder and spin-coated film states are almost the same, which are 15 ns and 14 ns, respectively. The calculated *k*_r_s are 4.27 × 10^7^ s^–1^ and 4.57 × 10^7^ s^–1^, which are a little higher than that in cyclohexane solution (*k*_r_ = 3.08 × 10^7^ s^–1^). The *k*_nr_s are very small, only 2.40 × 10^7^ s^–1^ and 2.57 × 10^7^ s^–1^, which are about half of that in cyclohexane (*k*_nr_ = 5.25 × 10^7^ s^–1^). Hence, the fluorescence enhancement from the solution to the solid state is due to the suppression of the nonradiative decay process. This is reasonable considering the relatively high flexibility of the [(2-dimethylboryl)phenyl]ethynyl moiety, the rotation of which should be restricted in the solid state and thus effectively suppress the nonradiative decay process. The moderately high *Φ*_F_s of ***m*-BPhANPh_2_-Cp** in solution and very high *Φ*_F_s in the solid state demonstrate its promising emission properties.

**Figure 4:**
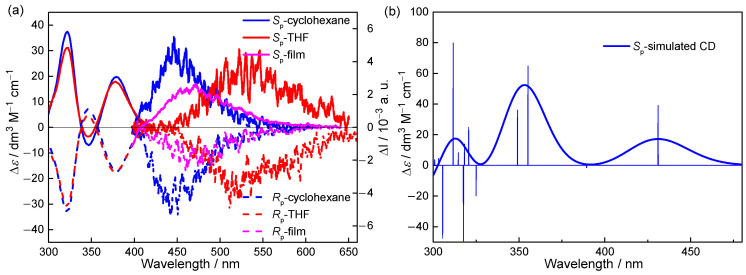
(**a**) CD (c = 3.0 × 10^–5^ M), and CPL (c = 3.0 × 10^–5^ M, *λ*_ex_ = 380 nm) spectra of ***m*-BPhANPh_2_-Cp** in cyclohexane, THF and film. (**b**) The theoretically simulated CD spectra of **(*S*_p_)-*m*-BPhANPh_2_-Cp,** calculated at the TD-PBE0/6-31G(d) level.

Encouraged by the promising fluorescence properties of ***m*-BPhANPh_2_-Cp**, we next prepared its enantiomerically pure form and explored the chiroptical properties (Figure 4, Table 2). The enantiomers of ***m*-BPhANPh_2_-Cp** were prepared through supercritical fluid chromatography (SFC) on a chiral column. The obtained enantiomers have very high optical purity, with the enantiomeric excess (*ee*) higher than 99% (Table 3). The configuration was determined by the comparison between the experimental and theoretically simulated ECD spectra of *S*_p_-isomer. The *R*_p_-isomer and the *S*_p_-isomer of ***m*-BPhANPh_2_-Cp** correspond to the first (*t*_R_ = 2.479 min) and the second (*t*_R_ = 3.224 min) elution fraction, respectively. It was found that the CD spectra exhibit perfect mirror-image Cotton effect with large ∆*ε*. In cyclohexane, the *S*_p_-isomer shows intense positive CD signals at 322 nm and 379 nm. The longest wavelength CD band is assignable to the S_0_→S_1_ transition, and the corresponding absorbance dissymmetry factor (*g*_abs_) was determined to be 7.57 × 10^–3^. In addition, the CD spectra are not affected by the solvent. From cyclohexane to THF, no obvious changes were observed in the CD spectra. More importantly, intense mirror-image CPL signals were also detected in the solution for ***m*-BPhANPh_2_-Cp**. In cyclohexane, the *S*_p_-isomer of ***m*-BPhANPh_2_-Cp** exhibits a positive signal with the *g*_lum_ at the maximum fluorescence wavelength (456 nm) up to +9.62 × 10^−3^. Similar to the emission spectra, the CPL signal in THF also shows a significant red shift compared to cyclohexane, and the *g*_lum_ at the maximum emission wavelength (536 nm) is as high as +8.29 × 10^−3^. The |*g*_lum_|s are very high values among small organic molecules, especially in dilute solution. Due to high *ε* and |*g*_lum_|, the *B*_CPL_s in cyclohexane and THF can reach 56.7 and 26.6 M^−1^ cm^−1^ in spite of their moderate *Φ*_F_. The *B*_CPL_s of ***m*-BPhANPh_2_-Cp** are also very high for SOMs with such a simple structure. Therefore, utilizing the CT nature of ***m*-BPhANPh_2_-Cp**, it is possible to tune the CPL from blue to yellow just by changing the solvent. Moreover, intense CPL signals were also observed in the spin-coated film state with |*g*_lum_| up to 5.40 × 10^−3^. Considering both high *Φ*_F_ and high |*g*_lum_| of ***m*-BPhANPh_2_-Cp** in the film state, it is expected to be an excellent solid CPL-emitter. So far, most CPL emitters can show intense CPL in either solution or solid state, the examples with intense CPL in both solution and solid state are still very rare.

## 3. Conclusions

In summary, we have disclosed a unique triarylborane-based [2.2]paracyclophane derivative, ***m*-BPhANPh_2_-Cp**, in which the pseudo-*meta* position was modified with the electron-accepting [(2-dimethylboryl)phenyl]ethynyl and electron-donating N,N-diphenylamino. Due to the intramolecular CT feature of S_1_ state, the fluorescence of this compound is tunable from blue in cyclohexane to yellow in THF with moderate *Φ*_F_s (0.15–0.37). In addition, this molecule can emit bright blue fluorescence in solid state with *Φ*_F_s up to 0.64 in both powder and spin-coated film states. The fluorescence enhancement from the solution to the solid state is in part ascribed to the suppressed nonradiative decay as the result of restricted intramolecular rotation. Moreover, the bulky steric effect and strong electronic effect of diphenylamino and BMes_2_ substituents are also important to avoid the ACQ effect since the bulky steric effect can effectively suppress the intermolecular π–π interactions in the solid state, and the electronic effect can induce CT emission with large Stokes shift to prevent self-absorption in the solid state. Most notably, the enantiomeric forms of ***m*-BPhANPh_2_-Cp** can show strong CPL signals in both dilute solution and solid state with |*g*_lum_|s up to 9.62 × 10^−3^ and 5.40 × 10^−3^, respectively. Thus, it is possible to achieve tunable CPL from blue to yellow in solution with high *B*_CPL_s ranging from 56.7 to 26.6 M^−1^ cm^−1^ and intense blue CPL combing high *Φ*_F_ and |*g*_lum_| in the solid state. The intense CPL of ***m*-BPhANPh_2_-Cp** in both solution and solid state implies its great potential uses as an excellent CPL emitter.

## 4. Materials and Methods

### 4.1. Reagents and Instruments

**Reagents:** All reactions were carried out under a nitrogen atmosphere. The starting material 4-Iodo-15-(*N,N*-diphenyl)amino [2.2]paracyclophane (***m*-INPh_2_-Cp**) was prepared according to the literature [43].

PdCl_2_(PPh_3_)_2_ and CuI were purchased from Energy Chemical (Shanghai, China), Et_2_NH was purchased from Macklin Biochemical Technology Co., Ltd (Shanghai, China)., *n*-BuLi was purchased from Shangyu Hualun Chemical Industry Co., Ltd (Shaoxing, China).

**Instruments:** Melting points (M.p.) were determined on a Tektronix XT-4 instrument (Beijing, China). ^1^H and ^13^C NMR spectra were measured on a Bruker 400 spectrometer (Berlin, Germany). High-resolution mass spectra (HRMS) were obtained on an Agilent electrospray ionization time-of-flight (ESI-TOF) mass spectrometer (Santa Clara, CA, USA). UV-vis absorption and fluorescence spectra were recorded with a Hitachi U-2910 spectrometer and a Hitachi F-7000 spectrometer, respectively (Tokyo, Japan). CD and CPL spectra measurements were performed on an Applied Photophysics Chirascan (Surrey, UK) and Jasco CPL 300 spectrometer (Tokyo, Japan). The transient fluorescence decay data was determined using an Edinburgh Instrument FLS920 spectrometer (Edinburgh, UK).

### 4.2. Computational Methods

For DFT calculations, the PBE0 functional and 6-31G(d) basis set were adopted in the Gaussian 16 software package [51]. The ground state geometries were optimized using the presumable structure as the initial structure. To ensure the lowest energy for the computational optimized structure, all the geometry optimizations were accompanied by a frequency calculation and ensured that only positive frequencies were obtained. Based on the optimized structure of the ground state, the vertical transitions were further calculated using the time-dependent DFT (TD-DFT) method. The frontier molecular orbitals were visualized through GaussView 6.0 software. The calculated UV−vis absorption and simulated CD spectra were generated by SpecDis 1.71 with a sigma of 0.16 eV [52].

### 4.3. Synthetic Methods

**4-(2-Bromophenylethynyl)-15-(*N,N*-diphenyl)amine[2.2]paracyclophane (*m*-BrPhANPh_2_-Cp):** To a mixture of 4-Iodo-15-(*N,N*-diphenyl)amino[2.2]paracyclophane (***g*-INPh_2_-Cp**) (413 mg, 0.824 mmol), (2-bromophenyl)acetylene (178 mg, 0.989 mmol), CuI (11 mg, 7% mmol) and PdCl_2_(PPh_3_)_2_ (28.8 mg, 5% mmol), Et_2_NH (12 mL) was added under a stream of nitrogen. The mixture reacted at room temperature for 12 h. After the reaction was finished, a saturated solution of NaCl was added, and the aqueous layer was extracted with ethyl acetate. The combined organic layer was dried over anhydrous Na_2_SO_4_, filtered, and concentrated under reduced pressure. The resulting mixture was subjected to a silica gel column chromatography (30/1 petroleum ether/ CH_2_Cl_2_, *R*_f_ = 0.60) to afford 228 mg (0.44 mmol) of ***m*-BrPhANMe_2_-Cp** in 63% yield as yellow solids. M.p.: 132.5–133.2 °C; ^1^H NMR (400 MHz, CDCl_3_): δ 7.64 (dd, *J* = 8.0, 0.8 Hz, 1H), 7.57 (dd, *J* = 7.6, 1.6 Hz, 1H), 7.35–7.28 (m, 5H), 7.21–7.15 (m, 5H), 7.11 (t, *J* = 7.2 Hz, 2H), 7.03 (d, *J* = 7.6 Hz, 1H), 6.94 (d, *J* = 7.6 Hz, 1H), 6.85 (d, *J* = 1.6 Hz, 1H), 6.34 (dd, *J* = 8.0, 2.0 Hz, 1H), 6.23 (dd, *J* = 7.6, 1.6 Hz, 1H), 5.81 (d, *J* = 1.6 Hz, 1H), 3.65–3.48 (m, 1H), 3.06–2.66 (m, 7H).; ^13^C NMR (100 MHz, CDCl_3_) δ 149.2, 146.5, 144.6, 143.4, 139.9, 139.3, 136.6, 133.9, 133.2, 132.5, 132.4, 131.9, 130.4, 129.3, 129.1, 128.2, 127.0, 125.9, 125.8, 125.5, 124.3, 123.9, 122.8, 94.2, 91.1, 34.9, 34.7, 33.8, 32.9; HRMS (ESI) *m*/*z*: [M+H]^+^ Calcd for C_36_H_29_BrN: 554.1478; Found 554.1477.

**4-(2-dimesitylborylphenylethynyl)-15-(*N,N*-diphenyl)amine[2.2]paracyclophane (*m*-BPhANPh_2_-Cp)**: To a solution of 4-(2-Bromophenylethynyl)-15-(*N,N*-diphenyl)amine[2.2]paracyclophane (***m*-BrPhANPh_2_-Cp**) (227 mg, 0.411 mmol) in anhydrous THF (15 mL), a hexane solution of *n*-BuLi (0.33 mL, 2.5 M, 0.82 mmol) was added dropwise by syringe at –78 °C. After the mixture was stirred at the same temperature for 1.5 h, a solution of dimesitylboron fluoride (275 mg, 1.03 mmol) in anhydrous THF (5 mL) was added to the reaction mixture via a syringe. Then, the reaction mixture was warmed to room temperature and stirred overnight. The reaction was quenched with a saturated solution of NaCl and the aqueous layer was extracted with ethyl acetate. The combined organic layer was dried over anhydrous Na_2_SO_4_, filtered, and concentrated under reduced pressure. The solvent was removed and purified by a silica gel column chromatography (5/1 petroleum ether/CH_2_Cl_2_, *R*_f_ = 0.45) to afford 235 mg (0.325 mmol) of ***m*-BPhANPh_2_-Cp** in 79% yield as yellowish solids. M.p.: 134.7–135.2 °C; ^1^H NMR (400 MHz, CDCl_3_): δ 7.57 (d, *J* = 7.2 Hz, 1H), 7.39 (td, *J* = 8.0, 2.4 Hz, 1H), 7.31 (s, 1H), 7.29–7.25 (m, 5H), 7.14 (d, *J* = 7.6 Hz, 4H), 7.09 (t, *J* = 7.2 Hz, 2H), 6.89 (d, *J* = 7.6 Hz, 1H), 6.82 (s, 4H), 6.58 (d, *J* = 7.6 Hz, 1H), 6.22 (dd, *J* = 7.6, 1.2 Hz, 1H), 5.98 (d, *J* = 1.6 Hz, 1H), 5.91 (d, *J* = 7.2 Hz, 1H), 5.75 (d, *J* = 1.6 Hz, 1H), 3.38–3.29 (m, 1H), 2.99–2.58 (m, 7H), 2.32 (s, 6H), 2.05 (s, 12H); ^13^C NMR (100 MHz, CDCl_3_) δ 149.6, 149.1, 146.3, 143.1, 142.8, 140.9, 139.6, 138.9, 138.7, 136.6, 134.4, 133.5, 132.8, 131.6, 131.4, 130.2, 130.0, 129.2, 128.4, 128.2, 127.7, 125.7, 125.4, 125.0, 123.8, 122.5, 93.8, 92.7, 34.9, 34.7, 33.7, 33.0, 23.3, 21.4; HRMS (ESI) *m*/*z*: [M+H]^+^ Calcd for C_54_H_51_BN: 724.4109; Found 724.4104.

### 4.4. Chiral SFC Separation Data

**Analytical separation method** of ***m*-BPhANPh_2_-Cp:**

Instrument: Waters UPC2 analytical SFC (SFC-H).
Column: Chiralpak AD-3 50 × 4.6mm I.D., 3 µm.
Mobile phase: A for CO_2_ and B for ethanol (0.05%DEA).
Gradient: B 5-40%.
Flow rate: 2.5 mL/min.
Back pressure: 100 bar.
Column temperature: 35 °C.

**Preparative separation method of *m*-BPhANPh_2_-Cp:**

Instrument: WATERS150 preparative SFC(SFC-26).
Instrument: WATERS150 preparative SFC(SFC-26).
Column: ChiralPak AD, 250 × 30mm I.D., 10 µm.
Mobile phase: A for CO_2_ and B for ethanol.
Gradient: B 25%.
Flow rate: 120 mL/min.
Back pressure: 100 bar.
Column temperature: 38 °C.
Cycle time: ~2.5 min.

## Figures and Tables

**Table 1:** Photophysical properties of ***m*-BPhANPh_2_-Cp**.

	*λ*_abs_/nm (*ε*/10^4^ M^−1^ cm^−1^)	*λ*_em_/nm	∆*ν*/10^3^ cm^−1^	*Φ*_F_	*τ*/ns	*k*_r_/10^7^ s^−1^	*k*_nr_/10^7^ s^−1^
cyclohexane	305 (3.19), 373 (0.91)	443	4.23	0.37 ^1^	12	3.08	5.25
toluene	306 (3.34), 369 (1.00)	473	5.96	0.28 ^1^	−	−	−
chloroform	305 (3.17), 372 (0.85)	513	7.39	0.16 ^1^	−	−	−
THF	305 (4.28), 368 (1.38)	527	8.20	0.15 ^1^	−	−	−
film	−	465	−	0.64 ^2^	15	4.27	2.40
powder	−	473	−	0.64 ^2^	14	4.57	2.57

^1^ Calculated using quinine sulfate as a standard. ^2^ Determined by a calibrated integrating sphere.

**Table 2:** Chiroptical properties of ***m*-BPhANPh_2_-Cp**.

	*R*_p_-Isomer			*S*_p_-Isomer		
	*g*_abs_/10^−3^ (*λ*/nm)	*g*_lum_/10^−3^ (*λ*/nm)	*B*_CPL_/ M^−1^ cm^−1^	*g*_abs_/10^−3^ (*λ*/nm)	*g*_lum_/10^−3^ (*λ*/nm)	*B*_CPL_/ M^−1^ cm^−1^
cyclohexane	−7.27 (380)	−9.13 (454)	53.7	+7.57 (379)	+9.62 (456)	56.7
THF	−6.03 (378)	−7.23 (531)	23.1	+6.51 (378)	+8.29 (536)	26.6
film	−	−4.32 (466)	−	−	+5.40 (466)	−

**Table 3:** The SCF profiles of ***m*-BPhANPh_2_-Cp**.

	Fraction	Retention Time/min	Area	Area%	*ee*
*rac*-form	*R*_p_-isomer	2.427	2,190,497	50.6	
	*S*_p_-isomer	3.185	2,141,703	49.4	
*R*_p_-isomer	*R*_p_-isomer	2.479	7,241,036	100	>99%
	*S*_p_-isomer	−	−	−	−
*S*_p_-isomer	*R*_p_-isomer	2.476	6226	0.08%	−
	*S*_p_-isomer	3.224	7,993,768	99.92%	>99%

## Data Availability

The data presented in this study are available in the paper.

## Supplementary Materials

The following supporting information can be downloaded at: https://www.mdpi.com/article/10.3390/molecules30020390/s1. Figure S1: Chiral SCF profiles of (a) **rac-*m*-BPhANPh_2_-Cp,** (b) ***R*_p_-(-)-*m*-BPhANPh_2_-Cp,** (c) ***S*_p_-(+)-*m*-BPhANPh_2_-Cp**.; NMR Spectra of ***m*-BrPhANPh_2_-Cp** and ***m*-BPhANPh_2_-Cp**; total energies and cartesian coordinates of the optimized structure.
