# Supplementary material for: [(2-Dimesitylboryl)phenyl]ethynyl-Substituted [2.2]Paracyclophane Exhibiting Circularly Polarized Luminescence in Both Solution and Solid-State
Source: Molecules. 2025 Jan 18;30(2):390. doi: 10.3390/molecules30020390 (PMC11767752; doi:10.3390/molecules30020390)
Supplement: Supplementary file 1 [file molecules-30-00390-s001.zip › molecules-3398183-supplementary.pdf]

Supporting Information

[(2-Dimesitylboryl)phenyl]ethynyl-Substituted [2.2]Paracyclophane Exhibiting Circularly Polarized Luminescence in both Solution and Solid-State

LianFeng Guo[1], Mengyuan Zhang[2,*], Cui-Hua Zhao[1,*]

1 School of Chemistry and Chemical Engineering, Shandong University, Jinan 250100, P. R. China.

2 College of Chemical Engineering and Environmental Engineering, Weifang University, Weifang 261061, P. R. China.

E-mail: chzhao@sdu.edu.cn (C.Z.); zmywf2021@163.com (M.Z.)

Table of Contents

Optical Resolution of ***m*-BPhANPh$_2$-Cp** S3

NMR Spectra S4

Total Energies and Cartesian Coordinates of the Optimized Structure S6

Optical Resolution of ***m*-BPhANPh$_2$-Cp**

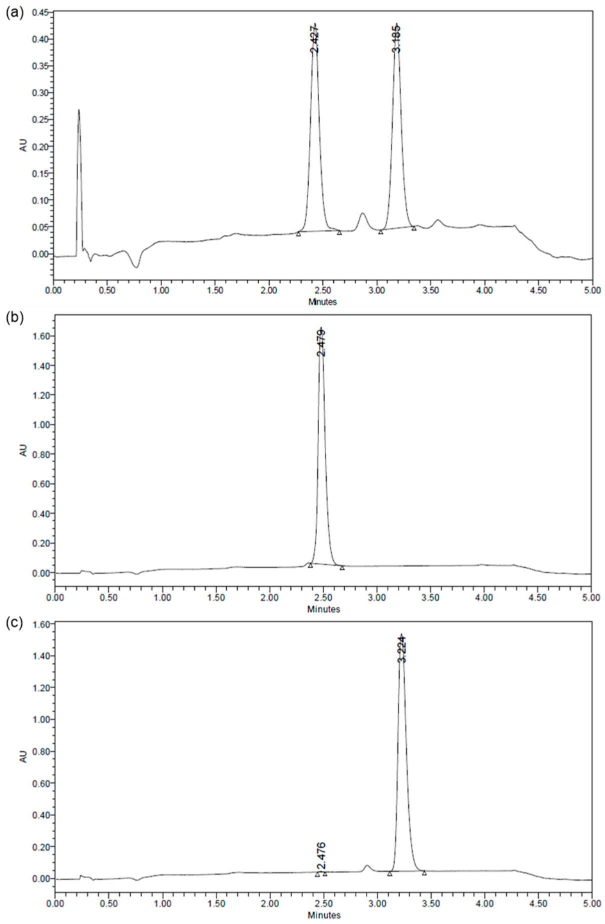

Figure S1. Chiral SCF profiles of (a) **rac-*m*-BPhANPh$_2$-Cp,** (b) ***R*$_p$-(-)-*m*-BPhANPh$_2$-Cp,** (c) ***S*$_p$-(+)-*m*-BPhANPh$_2$-Cp**.

NMR Spectra

^{1}H NMR of ***m*-BrPhANPh$_2$-Cp** (400 MHz, $CDCl_3$, rt)

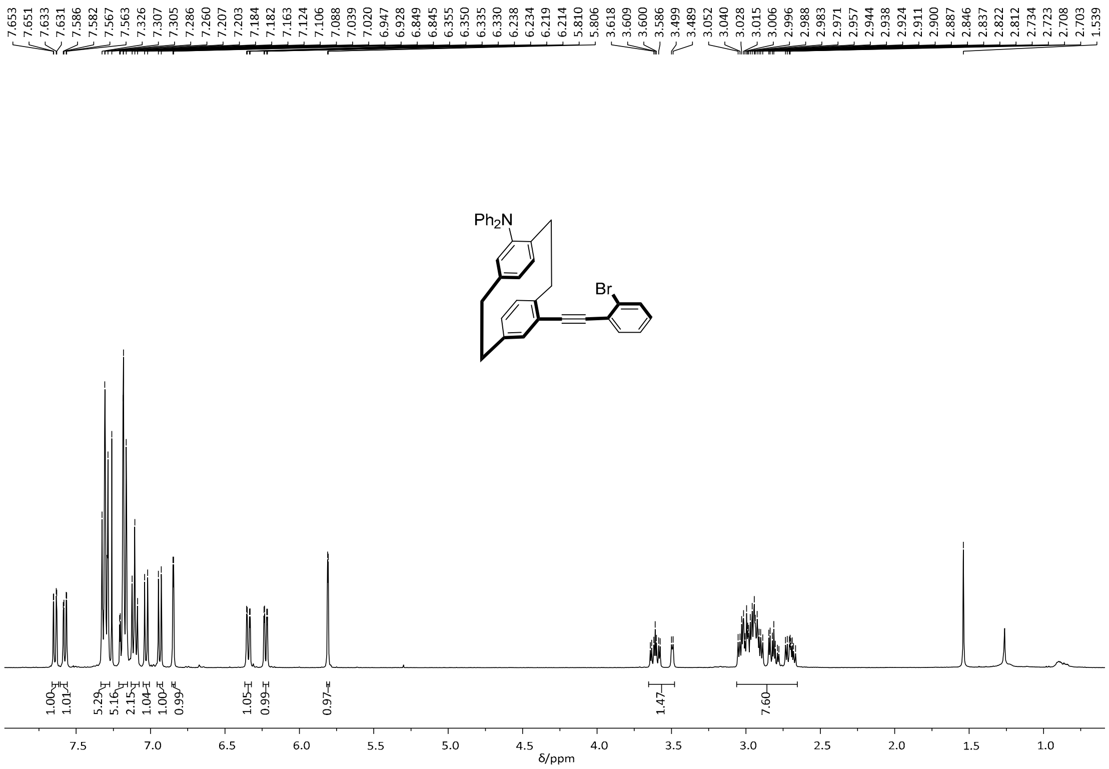

^{13}C NMR of ***m*-BrPhANPh$_2$-Cp** (100 MHz, $CDCl_3$, rt)

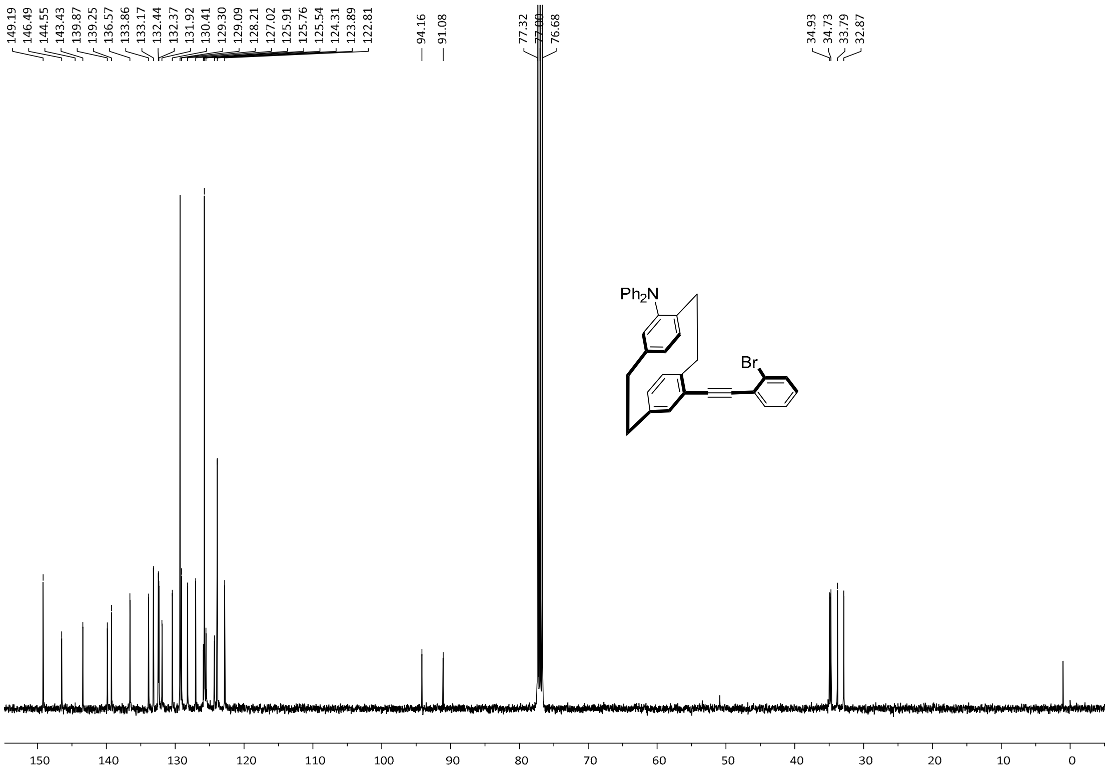

^{1}H NMR of ***m*-BPhANPh$_2$-Cp** (400 MHz, CDCl$_3$, rt)

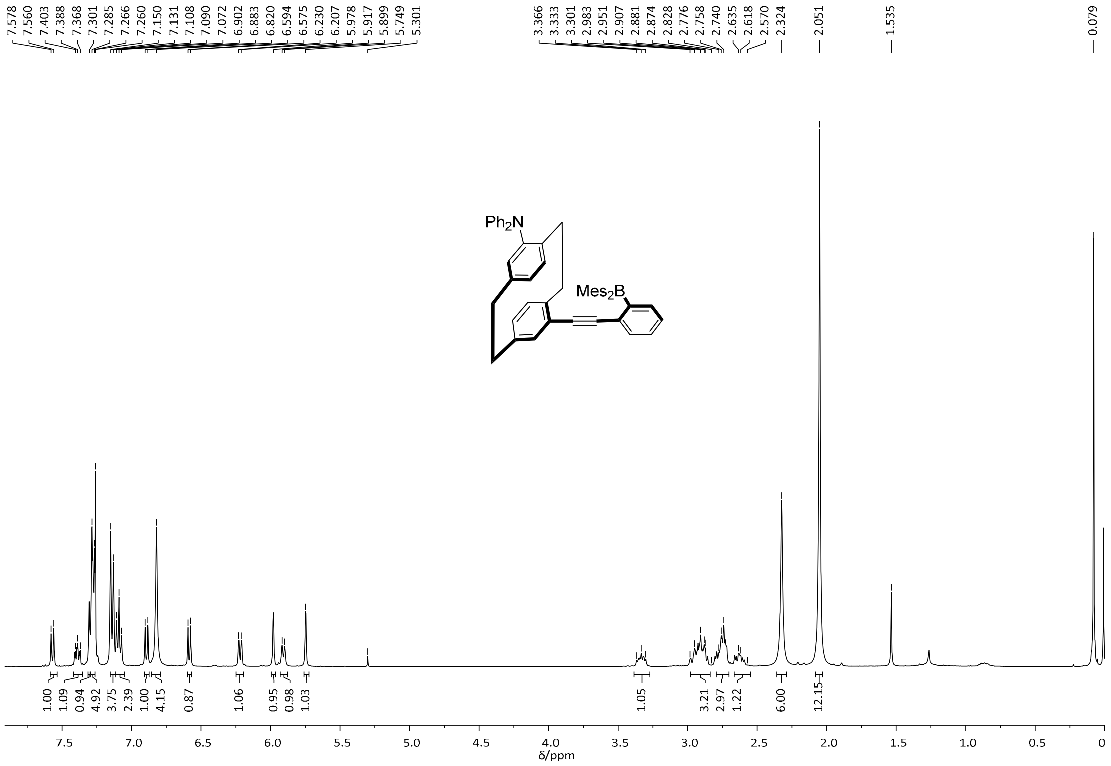

^{13}C NMR of ***m*-BPhANPh$_2$-Cp** (100 MHz, CDCl$_3$, rt)

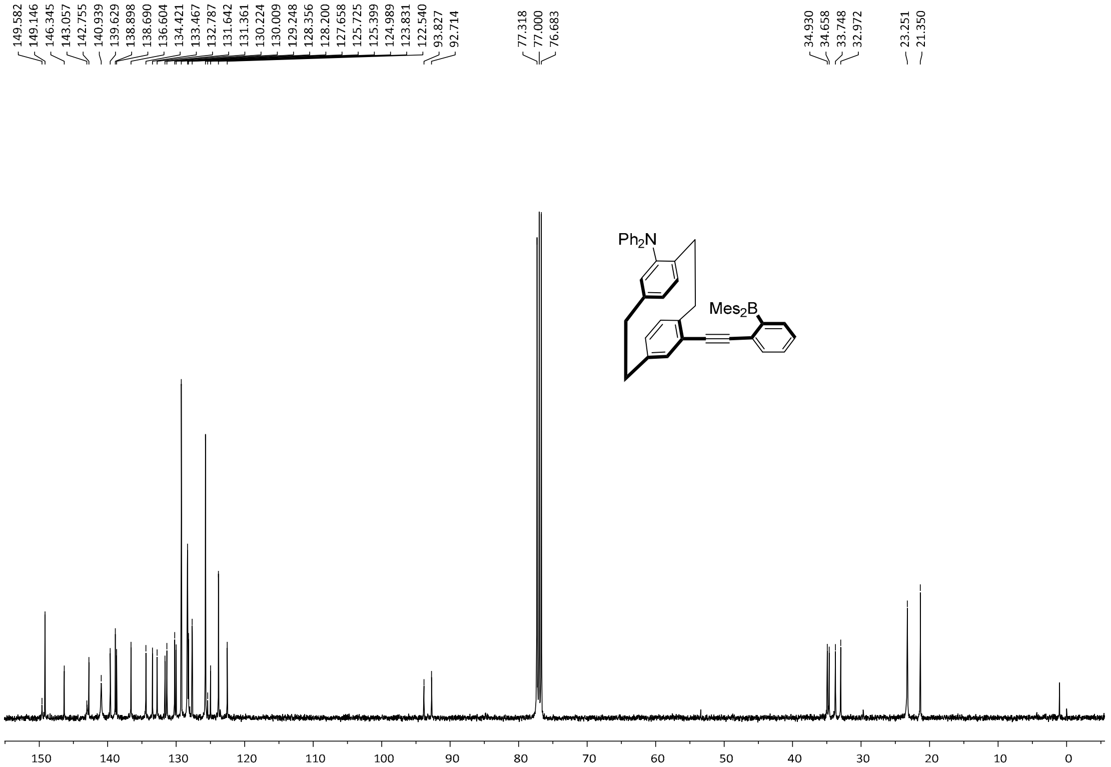

Total Energies and Cartesian Coordinates of the Optimized Structures

m-BPhANPh$_2$-Cp in S$_0$ State

Total energy (PBE0/6-31g(d)) -2164.79099966 a. u.

	X	Y	Z
C	0.79176500	0.80819700	-1.11468000
C	1.73039400	1.51496100	-0.79434000
C	-0.29152900	-0.03837100	-1.47598700
C	-0.19200500	-1.41728100	-1.20963700
H	0.70239700	-1.77674300	-0.70665800
C	-1.28307000	-2.26206100	-1.38780600
C	3.52247600	-0.49733300	1.00238600
C	8.51051100	-0.73483000	-1.08497500
C	-2.46478200	-0.40725800	-2.40321600
H	-3.37627400	-0.02038800	-2.85503500
C	-2.35805300	-1.76492200	-2.13341000
H	-3.18458500	-2.42756600	-2.38194000
C	-1.51822700	0.49991200	-1.92869300
C	2.93953100	-0.11317400	2.23590100
C	3.30603100	-1.82171300	0.55032400
C	6.31542600	0.19438200	-1.59448200
C	-3.08373600	0.80582700	0.37788700
C	2.00822700	-2.34732000	2.54819000
C	2.54989200	-2.71043500	1.31700200
H	2.39337000	-3.72288200	0.94658400
C	-4.14077300	-0.13499800	0.38734900
C	5.90753300	0.03715900	-0.24815200
C	3.08988800	1.27412900	2.81011400
H	4.12199400	1.63790200	2.77687500
H	2.76504500	1.29266300	3.85522300
H	2.48332600	2.00162400	2.25996300
C	6.83291200	-0.51084300	0.67130300
C	5.39974900	0.76992300	-2.64748300
H	4.36662500	0.42122000	-2.54739500
H	5.74541500	0.49593800	-3.64957200
H	5.36936900	1.86430900	-2.59427400
C	4.06892700	1.98189900	-0.07796700
C	5.05548600	2.96145700	0.11878800
H	6.05661500	2.63890800	0.39615500
C	-1.91658700	0.44261200	1.04709100
C	-1.92664800	1.92328000	-1.63601200
H	-2.29143300	2.43263500	-2.53670700

H	-1.04913000	2.47993500	-1.29353000
C	7.59124800	-0.20621800	-1.98924200
H	7.87796200	-0.09673400	-3.03441200
C	2.78348800	2.42257300	-0.48877700
C	8.11267600	-0.86666200	0.24363200
H	8.81823800	-1.26608000	0.97083700
C	6.49406200	-0.71057200	2.12800800
H	6.08001600	0.19435700	2.58661600
H	7.38775300	-0.98693900	2.69653900
H	5.74994500	-1.50253100	2.26391300
C	-1.65657000	-0.87861600	1.39329500
H	-0.66917900	-1.14458700	1.76412200
C	2.21353000	-1.03930600	2.98470900
H	1.79716300	-0.72960600	3.94238200
C	9.87549800	-1.17434000	-1.53491400
H	9.85760800	-2.21179300	-1.89336900
H	10.60223700	-1.12527000	-0.71766000
H	10.24555800	-0.55404100	-2.35795600
C	1.24756100	-3.33176000	3.39191900
H	1.01628800	-4.24434800	2.83341300
H	0.30465100	-2.90638700	3.75469700
H	1.82769100	-3.62501800	4.27594300
C	2.53245300	3.79534100	-0.65841200
H	1.54761700	4.11040500	-0.99128100
C	-3.86060400	-1.47227500	0.69427700
H	-4.63652900	-2.21928900	0.54837900
C	-3.06772300	2.00774700	-0.54229500
H	-2.94286900	2.94886000	0.00776400
H	-4.03357900	2.07780000	-1.04904000
C	3.83781400	-2.31202800	-0.77290000
H	3.51595500	-1.66964100	-1.60075900
H	3.47549400	-3.32323100	-0.98402600
H	4.93184800	-2.33473600	-0.78935500
C	3.52230200	4.73615300	-0.41060600
H	3.30477200	5.79354700	-0.53671500
C	-2.57081200	-1.87578300	1.05248300
C	-1.44709000	-3.53225800	-0.59352200
H	-0.48232400	-4.03459500	-0.46162600
H	-2.09461000	-4.22587600	-1.14151700
C	4.79288200	4.32031100	-0.01950100
H	5.57762900	5.04992500	0.16160600
B	4.46319100	0.48645600	0.20765200
C	-2.08299400	-3.28768800	0.84910800
H	-2.89766700	-4.00670300	0.99077600

H	-1.31870600	-3.52116700	1.59841100
H	-1.11273500	1.17114900	1.13525200
N	-5.44427500	0.26635800	0.00036800
C	-5.99314100	1.45886100	0.53534200
C	-5.61436100	1.95014300	1.79021700
C	-6.94502300	2.16845000	-0.20969500
C	-6.16874200	3.12971500	2.27640500
H	-4.88238600	1.40726400	2.37916900
C	-7.50373200	3.33715800	0.29172400
H	-7.24179900	1.79240800	-1.18462700
C	-7.11693300	3.83031900	1.53606800
H	-5.86277300	3.49458900	3.25362700
H	-8.23897900	3.87294400	-0.30315600
H	-7.54884300	4.74889000	1.92281800
C	-6.35751600	-0.68371700	-0.53051300
C	-6.06800100	-1.35283500	-1.72350100
C	-7.56064100	-0.96229400	0.12755400
C	-6.95819800	-2.28607900	-2.24249500
H	-5.13995800	-1.12922100	-2.23709400
C	-8.45500400	-1.88467100	-0.40471100
H	-7.78930400	-0.44703900	1.05574700
C	-8.15868100	-2.55482700	-1.58899300
H	-6.71819600	-2.79414800	-3.17301500
H	-9.38549000	-2.08844400	0.11872600
H	-8.85776200	-3.27729100	-2.00060300

***m*-BPhANPh$_2$-Cp in S$_1$ State**

Total energy (PBE0/6-31g(d)) - 2164.84905605 a. u.

	X	Y	Z
C	0.49359000	0.56440800	-1.34622500
C	1.36369000	1.35507400	-0.99384600
C	-0.51295300	-0.35632100	-1.69365400
C	-0.34582000	-1.73842700	-1.42915600
H	0.56845500	-2.04834800	-0.92737500
C	-1.38057200	-2.65081300	-1.61155900
C	3.27866300	-0.60017800	0.67719200
C	8.55176900	-0.30415200	-0.35070800
C	-2.70265200	-0.87791000	-2.55916000
H	-3.62866600	-0.53402400	-3.02011600
C	-2.51203100	-2.24030300	-2.32927800
H	-3.29375100	-2.95443700	-2.58139700
C	-1.79177900	0.08312400	-2.12068600
C	2.51850000	-0.39905600	1.85580700

C	3.30043500	-1.91421700	0.14445500
C	6.42596800	0.42650100	-1.30073400
C	-2.69866900	0.57271200	0.48813500
C	1.95024800	-2.77312200	1.98740700
C	2.64291700	-2.96295800	0.79141600
H	2.69414700	-3.96277900	0.35901500
C	-3.89355800	-0.22668500	0.47297600
C	5.76335200	0.26108300	-0.05807900
C	2.38187700	0.96230500	2.48646700
H	3.35040000	1.45863200	2.60492500
H	1.90791100	0.89273900	3.47218400
H	1.78059900	1.63239000	1.86009200
C	6.54649800	-0.19145400	1.03322800
C	5.70317900	0.90645100	-2.53496500
H	4.70016000	0.47674200	-2.61740600
H	6.26630300	0.64713400	-3.43865900
H	5.56879800	1.99464900	-2.52437100
C	3.68929500	1.96146400	-0.28699700
C	4.59973300	3.05058900	-0.20488300
H	5.62740700	2.82204100	0.06986900
C	-1.58566300	-0.03175900	1.05169300
C	-2.25176100	1.49377400	-1.86710100
H	-3.19917400	1.65961600	-2.39390900
H	-1.53323400	2.22945200	-2.24581800
C	7.78757500	0.13834700	-1.42605900
H	8.26428100	0.26491600	-2.39812500
C	2.34743800	2.31533400	-0.69495700
C	7.90760600	-0.45431800	0.87571900
H	8.48378500	-0.78416800	1.74025200
C	5.95391800	-0.37342900	2.40723900
H	5.40887300	0.52075900	2.73020300
H	6.73807500	-0.57159400	3.14625200
H	5.24181200	-1.20509300	2.43800300
C	-1.51035700	-1.41605500	1.21809800
H	-0.56263000	-1.84617400	1.52950200
C	1.88853100	-1.47440900	2.49102900
H	1.35536800	-1.29326300	3.42542900
C	10.01048200	-0.63391000	-0.50973200
H	10.16268600	-1.70734300	-0.68552400
H	10.58151900	-0.37131100	0.38794800
H	10.45148400	-0.10034500	-1.35854400
C	1.33567100	-3.93196000	2.72598000
H	0.94457800	-4.69116400	2.03761200
H	0.51662000	-3.60902700	3.37945500

H	2.07323400	-4.43513800	3.36525600
C	1.99502000	3.67231900	-0.90214100
H	0.97585900	3.89871400	-1.20835400
C	-3.79231800	-1.63627500	0.60122200
H	-4.68495200	-2.24052500	0.47870200
C	-2.46922500	1.82134500	-0.33494600
H	-1.55850200	2.28987400	0.04776300
H	-3.26946200	2.56058200	-0.25288400
C	4.00712300	-2.21329600	-1.15107900
H	3.64413300	-1.55963400	-1.95319200
H	3.84144300	-3.25091100	-1.46124600
H	5.08642500	-2.04751300	-1.07158800
C	2.91304800	4.68867300	-0.75593300
H	2.61928400	5.72173800	-0.92266800
C	-2.57372000	-2.25122200	0.82713600
C	-1.41755700	-3.89893600	-0.76482800
H	-0.40362300	-4.14206200	-0.43110600
H	-1.79259400	-4.76719800	-1.31952900
C	4.24347400	4.36654300	-0.41302700
H	4.98445900	5.15520500	-0.30558400
B	4.19896700	0.55910100	0.08122400
C	-2.33981300	-3.70644700	0.50851400
H	-3.30750100	-4.18787400	0.33065000
H	-1.87644600	-4.22448800	1.35608300
H	-0.67042700	0.54162700	1.15844600
N	-5.13713900	0.36075100	0.31151200
C	-5.36477600	1.67330100	0.79535200
C	-4.84217900	2.06512800	2.03361900
C	-6.12819100	2.56744200	0.03423800
C	-5.06463000	3.35709800	2.49147600
H	-4.27743900	1.35469300	2.62822800
C	-6.34139600	3.85521800	0.50346400
H	-6.52242700	2.25330300	-0.92708700
C	-5.80918500	4.25501900	1.72968300
H	-4.66158900	3.65960200	3.45296100
H	-6.91605300	4.55480700	-0.09573100
H	-5.97785000	5.26469300	2.09121400
C	-6.26184600	-0.35222300	-0.17965300
C	-6.14469300	-1.16138400	-1.31544700
C	-7.49571800	-0.22852800	0.47157400
C	-7.25970300	-1.83576900	-1.79461400
H	-5.18447100	-1.24718900	-1.81080100
C	-8.60188400	-0.90942800	-0.01747200
H	-7.57562500	0.38625700	1.36231500

C	-8.48969800	-1.71246800	-1.15120900
H	-7.16725000	-2.45412400	-2.68227800
H	-9.55445200	-0.81886100	0.49565400
H	-9.35835300	-2.24139800	-1.53136500
